# The Use and Abuse of Nursing Congresses

**Published:** 1909-07-17

**Authors:** 


### Nursing Administration.

### THE USE AND ABUSE OF NURSING CONGRESSES.

The advantages of those professional gatherings, which have grown so popular in recent years, lie on the surface. Workers in the same profession too often bury themselves in absorption in daily routine, and lose touch altogether with the lives and labours of fellow workers. They either grow satisfied with imperfect methods, or enjoy some system perfected at the cost of many years' endeavour in splendid isolation. When they can be brought together, and induced to compare results with people working in other places under absolutely different conditions their horizon is inevitably widened, their minds are opened to the benefits which may be obtained by giving and receiving information, the crust of indifference or superiority breaks away. No congress ever revolutionises opinion. It is sufficient if it prove the occasion for diverse thoughtful papers, if it provoke lively discussion, if it lift into view defects known only to the inner few, and point the way to necessary reforms, it may be in the far future. These are the aims of such world-wide congresses as those of the British Medical Association, the English Church, the British Association. The habit of attending such meetings is growing among us. They are commonly enlivened by festive gatherings which appeal alike to the serious and the frivolous. Every one can appreciate the delights of sitting with pleasant company in historic gardens, and eating strawberries to the strains of a military band. It may be that such an exercise does as much to enlarge the ideas as reiteration of well-worn theories in the heated lecture hall. It is certainly good both for host and guest that generous hospitality should be exercised towards those of different customs, countries and languages, that the kinship of common aims should make itself felt through all the distinctions of professions remarkable for their fine grades of precedence and qualification.

But the very attractiveness of such congresses renders them a ready vehicle in skilled hands for conveying erroneous impressions to the public. It has happened continually in various callings that meetings of an apparently representative character have been engineered for the glorification of some special group of individuals, or for the advertisement of some specious theory from which the main body of professional opinion strongly dissents. The interest and pleasure attaching to the gathering is made a cloak for the introduction of much to which the mass of individuals composing it object.

There can be no doubt that the nursing congress which will be held in London next week is being regarded by the great majority of English matrons and nurses with feelings varying between amused tolerance and settled disapproval. The presence of many foreign delegates of assured position and honourable notoriety, the concurrence of a Cabinet Minister and other distinguished people, even the adherence of some who have done admirable work in our own hospitals for the good of the nursing profession leave the main body of matrons and nurses absolutely without enthusiasm for the basis on which this imposing structure is reared. Their attitude was defined long ago by the most subtle of Latin poets, "We dread the Greeks even when they bring us gifts." It is a reasoned and reasonable dread. On what is it grounded?

In the first place English matrons have a justifiable dislike to the attempts which have been persistently made not only to force upon them a policy intensely distasteful to them, but even to make it appear that this policy is actually their own. They dissent strongly from the proposals for State registration of nurses put forward by the body which is responsible for summoning the present nursing congress, and they resent very deeply the imputations cast upon their sense and principles—imputations through which the supporters of registration in this particular form try to account for the small number of their own adherents. The matrons and nurses, who are constantly being told that their convictions are merely the outcome of cowardice, are not likely to be very enthusiastic about meetings in which every effort will be made to convince the public in this and other countries that the opinions they hold are not seriously entertained at all except by malicious and ignorant outsiders. They dread the glamour of festive gatherings which will be used as an illustration of an agreement in opinion which does not exist, and which ought not to exist, if by agreement is to be understood the domination of a small minority out of harmony with the general consensus of sound nursing opinion. In the second place English matrons distrust the whole tone and temper of the body controlling the congress. The most distressing manifestations of ordinary party politics have been freely imported into subjects which demand, above all else, the exercise of sound unbiased judgment. Those who touch upon these matters are constantly being drawn away, even it would seem in spite of themselves, into an unseemly heat; and some who in the calm of a philosophic mind perceive that the temper of the argument is of even greater importance than its matter, are driven to leave severely alone the conflict in which their motives are continually perverted.

We believe that it is the duty of those who love their profession to use the present occasion for the enunciation of sound principles, and to refute widely disseminated misrepresentations in respect to the general attitude of nurses towards the particular brand of legislative changes advocated by the promoters of the congress. A nursing congress is no place for recrimination and impatience with divergent views. It should have room for every shade of opinion, and those who may be called on to oppose will have ample opportunity for the exercise of a wise restraint and a good "temper" in discussion. The occasion calls for decisive action on the part of those well able to speak for the profession.

